# The Effectiveness of Prehospital Administration of Tranexamic Acid in Reducing Mortality in Trauma Patients: An Overview

**DOI:** 10.7759/cureus.49784

**Published:** 2023-12-01

**Authors:** Syed S Javeed, Mohammed A Altawili, Lujain Nadhem A Almubarak, Shoug A Alaodah, Mohannad Mastour A Alqarni, Omar I Odeh, Mohammed Ali B Asiri, Rakan Abdulrahman M Alotaibi, Arwa Ahmed A Alshammari, Zainab Adnan M Alqutayfi, Omniah Salem D Altemani, Dhafer Ahmed M Al Gharban, Zohair A Zafar

**Affiliations:** 1 Emergency Medicine, King Salman Armed Forces Hospital, Tabuk, SAU; 2 General Practice, Al-Aziziyah Primary Health Care Center, Tabuk, SAU; 3 Emergency Medicine, University of Sharjah, Sharjah, ARE; 4 Medical School, Qassim University, Qassim, SAU; 5 General Practice, King Abdullah Medical Complex, Jeddah, SAU; 6 Medical School, King Saud University College of Medicine, Riyadh, SAU; 7 General Practice, King Khalid University, Abha, SAU; 8 General Practice, Dar Al Uloom University, Al-Ahsa, SAU; 9 Emergency Medicine, University of Tabuk, Tabuk, SAU; 10 Emergency Medicine, King Abdulaziz University, Jeddah, SAU; 11 Medical School, King Abdulaziz University Hospital, Jeddah, SAU; 12 General Practice, Al-Awali Primary Health Care Center, Makkah, SAU

**Keywords:** antifibrinolytic, prehospital management, mortality rate, trauma, tranexamic acid

## Abstract

Tranexamic acid (TXA) is an antifibrinolytic drug that reduces bleeding by inhibiting plasminogen activation and fibrin clot degradation. Its role in prehospital trauma management remains unclear. This article aims to systematically review the current evidence on the effect of prehospital TXA administration on mortality in adult and pediatric trauma patients.

A literature search was conducted of PubMed, Web of Science, Scopus, and Cochrane databases from March 2023 to August 2023 for studies evaluating the impact of prehospital TXA use on trauma mortality. Inclusion criteria were articles published in the English language in the past 20 years focusing on clinical outcomes of prehospital TXA administration. Data on all-cause mortality, thromboembolic events, and time to TXA administration were extracted.

In adult trauma, prehospital TXA appears to reduce early all-cause mortality when given within three hours of injury without increasing thromboembolic risks. Some studies found decreased delayed mortality, while others found no difference. In pediatric trauma, preliminary evidence suggests TXA may lower in-hospital mortality in hemodynamically unstable patients, though higher doses may increase seizure risk.

Early prehospital administration of TXA within three hours of adult trauma may reduce mortality through improved hemorrhage control. Potential benefits in pediatric trauma warrant further investigation, balancing efficacy against safety risks such as seizures from high doses. Well-designed randomized trials are needed to validate optimal TXA dosing strategies across age groups and injury severity levels.

## Introduction and background

Tranexamic acid (TXA) is a competitive inhibitor of plasminogen activation, promoting blood clot formation. TXA is utilized to manage bleeding in various scenarios such as postsurgical procedures in patients with bleeding disorders such as von Willebrand’s disease, severe menstrual hemorrhage in women, tooth extractions, and ulcerative colitis. Additionally, it can prove beneficial in controlling bleeding following traumatic injuries [1]. There is a possibility for TXA to interact with blood clotting factors, which may subsequently lead to thrombosis. Other known side effects of TXA include nausea, vomiting, hypotension, visual disturbances, and urethral problems [1].

A meta-analysis conducted in 2020 demonstrated a reduced mortality rate and hemorrhagic expansion associated with the use of TXA in managing bleeding from traumatic brain injuries. Vasculo-occlusive events (pulmonary embolism, deep venous thrombosis, and myocardial infarction) were not statistically different between the groups that received TXA and the placebo group [2].

Major hemorrhage resulting from trauma is one of the leading causes of patient morbidity and mortality. It necessitates early and aggressive intervention to prevent further complications [3]. TXA plays a pivotal role in controlling hemorrhage [4]. It provides a potential method for managing major bleeding from various trauma sources, including intracerebral hemorrhage [4].

Several studies have demonstrated the beneficial role of TXA when administered within three hours of a primary insult in patients with traumatic brain injury. It has been shown to reduce mortality from post-trauma bleeding and decrease the risk of secondary brain injury due to the expansion of intracranial hemorrhage [2,5]. Conversely, some argue that the early use of TXA provides moderate or no benefits, while potentially causing moderate side effects [6].

This article reviews the potential role of prehospital administration of TXA in reducing mortality rates among trauma patients based on the latest data. It is an attempt to collate comprehensive evidence.

## Review

Methodology

This review article was conducted between March 1, 2023, and August 19, 2023. We searched online databases such as PubMed, Web of Science, Scopus, and Cochrane to find the latest studies that discussed the effect of prehospital use of TXA on a trauma patient’s mortality. We also incorporated some of the studies that discussed its use in hospitals, mostly in the emergency department, to draw a comparison between early and delayed use. We used the following keywords in our search strategy: “Tranexamic Acid,” “TXA,” “Trauma,” and “Mortality.” We limited our search to studies that were significant to our project, written in the English language, and published within the last 20 years.

Inclusion and Exclusion Criteria

We included all relevant studies published in the English language over the past 20 years that focused on the keywords mentioned above. We excluded certain types of studies, such as case reports, editorials, and cohort studies. On the other hand, we included randomized trials, meta-analyses, and systematic reviews. We excluded any studies or publications that did not primarily focus on the predefined keywords. We also excluded multiple studies and reviews as they did not meet our specific criteria.

Our group members evaluated the initial results and the methodologies of the surgical procedure by thoroughly reviewing the data. To ensure accuracy and minimize errors, we double-checked each member’s results.

Pharmacodynamics of tranexamic acid

TXA is a competitive inhibitor of plasminogen (the inactive form of plasmin) on the lysine receptors, thus exerting its antifibrinolytic activity by stabilizing the fibrin by preventing the binding of plasmin and plasminogen and degradation of fibrin. Fibrinolysis suppression can be manifested by a decrease in the blood levels of D-dimer in patients undergoing surgery. Compared to other synthetic antifibrinolytics such as ε-aminocaproic acid, TXA is six to ten times more potent. The antifibrinolytic activity of TXA is concentration-dependent [7,8].

Pharmacokinetics of tranexamic acid

With oral administration, maximum plasma concentration is reached within three hours, and gastrointestinal contents do not affect the pharmacokinetics of TXA [7]. The median peak concentrations have been reported to be 57.5, 34.4, and 12.8 mg L^−1^ for intravenous, intramuscular, and oral routes, respectively. The therapeutic concentration of 10 mg L^−1^ has been reported to be four minutes for the intramuscular route and 66 minutes for the oral route. For the intravenous route, the maximum concentration is reached at the end of the infusion which is much greater than 10 mg L^−1^. The bioavailability is 1.0 and 0.47 for the intramuscular and oral routes, respectively [9].

Uses of tranexamic acid

Using TXA controls gastrointestinal bleeding, reducing the odds of mortality by 16%, and by 20% if administered within the first three hours of bleeding [10].

TXA is also effective in treating menorrhagia and can be used in hemophilia patients undergoing minor procedures such as tooth extraction. It can be prescribed for angioedema cases and elective surgeries such as cardiac surgery. TXA is also useful in managing drug-induced bleeding, such as bleeding that may occur with tissue plasminogen activator administration. However, its use in trauma-induced bleeding is subject to debate [7,11].

Trauma-induced mortality

In 2017, the mortality from bleeding following a major injury dropped to 27%, a significant decrease from the 45% seen in 2008. Even though early mortality rates have dropped, exsanguination still remains the main cause of death. Regarding late deaths, traumatic brain injuries account for 52%, while multiple organ failures are responsible for 45%. The rate of red blood cell (RBC) transfusion reduced from 12 to 4, yet a high mortality of 48% persists when large-scale transfusions are involved [12].

Trauma-induced coagulopathy (TIC) is considered a separate disorder. TIC occurs after major trauma, which is a bleeding disorder due to alterations in fibrinolysis, endothelial, and platelet function. In fact, 61% of patients arriving at the hospital with an injury severity score over 45% have coagulopathy upon arrival. These coagulopathic patients have a four times higher risk of mortality compared to non-coagulopathic patients [13].

Tranexamic acid use in trauma patients in hospital

Two meta-analyses were conducted in 2020 [14,15] on the efficacy and safety of TXA use in traumatic brain injury. Both studies concluded that TXA lowers the mortality risk significantly when compared to control or placebo. TXA reduced the rate of hemorrhagic expansion and slightly reduced the risk of vascular-occlusive events (VOEs). There was no significant risk between the TXA and the control groups in the risk of myocardial infarction, pulmonary embolism, deep venous thrombosis, unfavorable outcomes (GOS), and the influence on neurosurgery and extracranial surgeries [2,16].

The World Maternal Antifibrinolytictrial (WOMAN) trial assessed the effect of TXA on mortality in patients with postpartum hemorrhage [17]. TXA showed a significant decrease in mortality when given early after postpartum hemorrhage, especially when administered in the first three hours after delivery [17]. All-cause death and hysterectomy were not reduced in the TXA group compared to the placebo group [17].

The Haemorrhage Alleviation With Tranexamic Acid-Intestinal System trial (HALT-IT trial) evaluated the effect of a high-dose 24-hour infusion of TXA versus placebo on gastrointestinal bleeding [18]. The study found that TXA did not reduce the risk of mortality or arterial thromboembolic events and that venous thromboembolic events (pulmonary embolism, deep venous thrombosis) were higher in the TXA group [18].

A meta-analysis of 64,724 patients revealed that using TXA decreased the odds of death by 16% when compared to patients who did not receive TXA. Moreover, if TXA was given within the first three hours, it was even more beneficial, reducing the odds of death by 20% compared to those who did not receive TXA in this early window. TXA has no difference in odds of VOEs compared to control [10]. A review article by Napolitano et al. discussed the use of TXA in trauma and provided a rational approach to TXA use in less than three hours after the severe hemorrhagic shock (systolic blood pressure 75 mmHg), with known fibrinolysis by TEG (LY30 3%), or with known predictors of fibrinolysis [19]. Based on data from the Clinical Randomisation of an Antifibrinolytic in Significant Haemorrhage 2 (CRASH-2 study), it was suggested that the decreased mortality may be attributed to bleeding control through the improvement of coagulation and prevention of clot lysis [20]. Other studies have suggested that improved survival with TXA may be due to inflammatory or immune effects.

Prehospital use of tranexamic acid in trauma patients

In an exploratory study examining the neuroprotective effects of TXA as part of the Clinical Randomisation of an Antifibrinolytic in Significant Head Injury 3 (CRASH-3) trial, the results indicated that TXA reduced the overall mortality rate by 20% in patients with various severities of traumatic brain injury. This reduction was likely due to a decrease in deaths caused by intracranial bleeding within the first 24 hours [21,22]. Traumatic brain injury patients often experience fibrinolysis which worsens intracranial bleeding. Pooled analysis of CRASH-2 and CRASH-3 studies for all-cause death at 28 days showed that TXA decreased its risk, but there was no difference in the risk of VOEs between the TXA and placebo groups.

Identical results were noted for all-cause death at 28 days when adding a study with prehospital use of TXA in isolated traumatic brain injury patients [21]. TXA is included in the prehospital care guidelines for trauma patients but not for isolated traumatic brain injury. The CRASH-3 study suggested that TXA can be used in isolated traumatic brain injury patients even if severely injured, as TXA is safe to use and even reduces head injury-related deaths when used within three hours of injury in patients with mild-to-moderate head injury but not in severe head injury [21,22].

Data from the National Center for Health Statistics was analyzed and showed that 3,409 and 2,236 deaths per year could have been prevented if TXA was administered within one hour and one to three hours after injury in trauma patients, respectively. Additionally, 1,371 deaths could have been averted if it had been administered to hypotensive or tachycardic trauma patients [23].

Overall, mortality risk was significantly lower in patients receiving prehospital TXA than in control traumatic patients. Thromboembolic events were also lower in the TXA group [24]. TXA was associated with smaller amounts of blood product transfusion but offered no superiority regarding the length of hospital or intensive care unit stay [25]. When prehospital TXA was given to bleeding trauma patients, it showed a significant reduction in 24-hour mortality, but no statistically significant difference was found in 28-30-day mortality nor in the VOEs [26]. Administering TXA to severely injured patients who require blood transfusion is advised, as well as monitoring patients for VOEs as TXA increases D-dimer and is a risk factor for VOEs [25,26].

In the context of prehospital resuscitation for hemorrhage-prone patients, it was observed that groups receiving either just prehospital red blood cells (pRBCs) or a combination of pRBCs and TXA had more severe injuries. This was evidenced by their higher median Injury Severity Scores (22 (10-34) for the pRBC group and 22 (17-36) for the pRBC + TXA group) compared to the groups that received only TXA (12 (5-21)) or no treatment at all (10 (4-20)) (p < 0.01) [27].

Regarding 30-day mortality rates, the pRBC + TXA and pRBC groups experienced higher mortality rates (18.2% and 28.6%, respectively) compared to the TXA group (6.6%) and the untreated group (7.4%). However, the combination of pRBC and TXA was associated with a 35% decrease in the risk of death within 30 days compared to the group receiving no treatment (hazard ratio = 0.65; 95% confidence interval = 0.45-0.94; p = 0.02) [27].

While the combination of pRBC and TXA did not improve survival rates within the first 24 hours, the pRBC group alone experienced a 61% decrease in the risk of death within the same timeframe compared to the untreated group (hazard ratio = 0.39; 95% confidence interval = 0.17-0.88; p = 0.02) [27].

Overall, the data suggest that there might be an additive effect when pRBC and TXA are used together. Therefore, it is recommended that TXA be administered to trauma patients who are also receiving prehospital pRBC transfusions [27].

Gulickx et al. [28] reported only 26% of prehospital TXA use in the Netherlands in severely injured patients. In another study by Van Wessem et al. [29], it was 49%, and only 10% in the English TARN database reported by Coats et al. [30]. This might be attributed to several factors mostly due to the short distance to the hospital or failure to identify severe hemorrhage as in internal bleeding and normotensive cases [28].

The most prevalent factor that affects prehospital TXA use is injury type. Patients who were more likely to receive TXA had higher Injury Severity Scores or airway intervention, with the site of the injury being the neck (possibly because it is a non-compressible area), and the mechanism of injury being road traffic accidents, penetrating trauma, and gunshots. Resources, protocols, skills, patient age and sex, priorities, consequences, and social influences also affected prehospital TXA administration [31].

The time of prehospital treatment is vital and is reflected in the overall survival rates. Every 15-minute delay decreases the survival rates by 10% until three hours. TXA appears to be more beneficial when administered early, with a 70% survival rate in immediate administration, and with no significant risk of increased VOEs [32].

Tranexamic acid administration in pediatric trauma patients

Children were excluded from the CRASH-2 and CRASH-3 studies. This was due to the complexity of conducting large-scale randomized controlled trials in emergency settings, particularly when the medication dosage is calculated based on body weight rather than being a fixed dose. While a fixed dose can simplify the process and minimize errors, it is not feasible to administer a fixed dose of TXA to children. Therefore, we cannot generalize the results from adult studies to the pediatric population [33].

Analyzing the use of TXA in different causes of bleeding patients, Shimizu et al. reported one child having a thrombotic adverse event in the TXA group and none in the control group [34]. In the other seven studies, there were no VOEs in either group. There were no seizures in the five studies in the seizure subgroup [35].

In a study, patients who were given TXA had an odds ratio of 5.39 compared to those who did not receive TXA, and the incidence rate was 2.7%. This rate increased when the dose of TXA was increased [36]. Of note, administering a high dose of TXA might increase the risk of seizures [35,36]. Hence, doctors suggest not using high doses of TXA.

Another study in Japan matched 1,914 pairs of patients. They found that seizures were more common in the group that received TXA. Out of 1,914 pairs, seven patients who received TXA had seizures, but none of the patients who did not receive TXA had seizures. This suggests that TXA might increase the risk of seizures in children [37].

However, when TXA was used in children with serious injuries from combat, it seemed to lower the risk of dying in the hospital, just like it did in adults. However, these children also received more blood products within the first 24 hours of treatment [38].

Some experts believe that a special type of test, called thromboelastographic-guided resuscitation, should be used to identify seriously injured or unstable children who might benefit from drugs that prevent blood clots from breaking down [39].

## Conclusions

This narrative review examined the current evidence on the efficacy and safety of prehospital TXA administration in adult and pediatric trauma patients. The findings indicate that early prehospital TXA use within three hours of injury in adults appears beneficial, as it is associated with reductions in early all-cause mortality without increasing thromboembolic risks.

Preliminary evidence in pediatric trauma suggests TXA may lower in-hospital mortality in hemodynamically unstable children. However, optimal dosing strategies remain unclear due to risks such as seizures from higher doses.

Overall, the evidence supports the consideration of prehospital TXA administration according to current guidelines. Nonetheless, additional high-quality research is needed. In particular, well-designed randomized controlled trials are warranted to validate dosing approaches across different age groups and injury severities. Furthermore, contextual factors influencing prehospital use and long-term outcomes beyond 28-day mortality require further exploration.

With more robust evidence, prehospital trauma teams can be better equipped to deliver TXA safely and maximize its potential lifesaving benefits in both adult and pediatric populations. Ongoing research efforts are important to continue developing trauma hemorrhage control strategies and improving survival outcomes following injury.
